# Effect of sodium deficiency on growth of surgical infants: a retrospective observational study

**DOI:** 10.1007/s00383-014-3619-2

**Published:** 2014-10-17

**Authors:** Fatima Mansour, Danielle Petersen, Paolo De Coppi, Simon Eaton

**Affiliations:** 1 Surgery Unit, UCL Institute of Child Health and Great Ormond Street Hospital for Children, 30 Guilford Street, London, WC1N 1EH UK; 2 Department of Dietetics, Great Ormond Street Hospital for Children, Great Ormond Street, London, WC1N 3JH UK

**Keywords:** Ileostomy, Sodium deficiency, Urinary sodium, Neonatal surgery, Growth

## Abstract

**Background/aim:**

Sodium is thought to be critical to growth. Infants who have an ileostomy may suffer from growth faltering, as sodium losses from stomas may be excessive. Urinary sodium measurements may indicate which patients could benefit from sodium supplementation; however, there is no consensus on what level of urinary sodium should be the cutoff for intervention. Our aim was to determine whether there is a relationship between urinary sodium and growth in infants undergoing ileostomy, colostomy and cystostomy.

**Methods:**

Following audit approval, a retrospective observational study of patient notes and chemical pathology data was carried out. All infants <1 year of age that had an ileostomy, colostomy, or cystostomy procedure between February 1997 and January 2014 were included. Patients’ weights, urinary and serum sodium and potassium levels and clinical variables were recorded until discharge. Weights were converted to *Z*-scores for analysis.

**Results:**

Forty patients were identified whose notes were available for review and who had at least three urinary sodium measurements. During their hospital stay, 11 (28 %) maintained weight within normal limits (*Z*-scores −2 to +2, 15 (38 %) were moderately malnourished (−3 to −2) and 14 (35 %) severely malnourished (<−3). Thirty patients had at least one urinary sodium <10 mmol/litre, six patients had their lowest recorded urinary sodium between 10 and 30 mmol/litre and only four patients had all their urinary sodium measurements >30 mmol litre. Electrolyte data were not normally distributed so that correlations between electrolytes and growth were tested using the non-parametric Spearman rank correlation coefficient. Urinary sodium levels positively correlated with growth (*r* = 0.3071, *p* < 0.0001), as did serum sodium levels (*r* = 0.2620, *p* = 0.0059) whereas there was no relationship between urine or serum potassium and growth.

**Conclusions:**

Poor growth is frequent in this group of patients and appears to be linked with sodium levels. Further work is necessary to draw up guidelines for appropriate sodium supplementation.

## Introduction

Sodium is the main cation of the extracellular fluid and is thus a key player in whole body fluid balance. There is therefore an obvious potential link between sodium and acute changes in weight, which are largely changes in fluid balance. During the first few days of life, infants lose extracellular fluid and sodium, and then shift from a negative to a positive sodium balance. However, a link between sodium and true somatic growth is less obvious. Although intracellular concentrations of sodium are low (potassium is the major intracellular cation), changes in cell mass and number (resulting in true growth) appear to be intimately linked with sodium availability [1]. This link has been shown in several experimental studies (reviewed [1]) in which true weight gain was shown to be linearly related to sodium intake. The biochemical basis for this effect of sodium on growth is unknown but may be mediated via intracellular pH.

In addition to experimental studies linking growth with sodium provision, there is evidence from several clinical studies that inadequate sodium supply impairs growth and that sodium supplementation restores growth. In particular, surgical infants with ileostomies are at risk of sodium depletion and impaired growth, which can be corrected by sodium supplement [2]. In addition, infants with cystic fibrosis [3], Pierre Robin sequence [4] and severe congestive heart failure [5] may have sodium depletion and benefit from sodium supplementation.

The optimum methods for assessing sodium adequacy would be either a 24-h urine collection, which allows sodium excretion to be directly compared with intake, or fractional sodium excretion (FENa), in which the sodium concentration in urine is corrected for variation in urine production. Low FENa indicates sodium retention by the kidney, suggesting decreased intake or sodium loss extrinsic to the urinary system such as sodium or volume depletion through gastrointestinal or skin loss. However, both these methods are too burdensome to be useful in routine monitoring of surgical infants, as they require either paired blood and urine samples (fractional sodium excretion) or a considerable burden on infants and/or nursing staff to ensure quantitative collection over 24 h.

In practice urinary sodium concentration usually measured from a spot urine sample; as this is a convenient and inexpensive method of monitoring sodium excretion. In line with the adult literature of patients with stomas [6], a 10 mmol/litre level of urinary sodium is often regarded as the lower limit of normal. The evidence base for this limit in surgical infants is limited, being based solely on the description of 11 patients in the report of Bower et al. [2]. However, recent data suggests that even surgical infants with urinary sodium <30 mmol/litre have impaired growth compared with those with urinary sodium >30 mmol/litre [7].

The aim of our study was to assess the relationship between urinary sodium and growth in infants with ileostomy, colostomy and cystostomy.

## Methods

This was a retrospective observational study on patients from Great Ormond Street Hospital for Children, operated between February 1997 and January 2014, with local hospital audit approval, of anthropometric (weight), biochemical and clinical data collected from post-surgical infants. Inclusion criteria were (i) infants <1 year of age at the time of surgery; (ii) ileostomy, colostomy or cystostomy performed; (iii) three or more urinary sodium measurements during initial admission; (iv) three or more weight measurements (plus birth weight). These patients were identified from theatre lists by OPCS codes and additional information was obtained from the notes and chemical pathology records.

Data collected (up to 1-year post-operatively, including outpatient visits) included: patient diagnosis, urinary and serum sodium and potassium levels, post-operative complications (infection, re-operation, haemodynamic comprise), weight, kidney disease, diuretics, length of ICU stay and length of hospital stay.

Urinary sodium and potassium levels were obtained from spot urinary tests carried out after the surgery. The lowest urinary sodium measurement for each patient was used to classify them using the following cut-offs: Group 1: >30 mM normal, Group 2: 10–30 mM deficient, Group 3: <10 mM severely deficient) [7].

The Microsoft Excel add-in LMS growth (version 2.69 [8]) was used to calculate *Z*-score values for raw weight measurements, using British 1990 growth reference [9]. The World Health Organisation [10] has suggested *Z*-score cut-offs to help diagnose growth faltering in weight <2, >−2 normal, <−2 moderate malnutrition, <−3 severe malnutrition. Once longitudinal data on weight was collected, they were converted to *Z*-weight for age scores and these cut-off values were used to assess if patients had at one point become moderately or severely malnourished.

Data are presented as mean ± SD, median (range) or *n* (%) as appropriate. Spearman’s non-parametric rank correlation coefficient was used to examine the relationship between urinary sodium and change in weight *Z*-score as the data did not meet the assumptions for the parametric equivalent; multi-level regression modelling (MLWin v2.29, Centre for MultiLevel Modelling, Bristol, UK) was used to examine changes in growth over time, estimates from the multilevel model are given as mean ± SEM.

## Results

During the time period Feb 1997–2014, 489 children underwent ileostomy, colostomy or cystostomy procedures. 274 were excluded because they were not infants <1 year of age at the time of the procedure, 141 further patients were excluded as they had fewer than three urinary sodium measurements. Of the remaining 74 patients, 40 had sufficient clinical data available and these infants formed the group that was studied.

### Weight and malnourishment

**Fig. 1:**
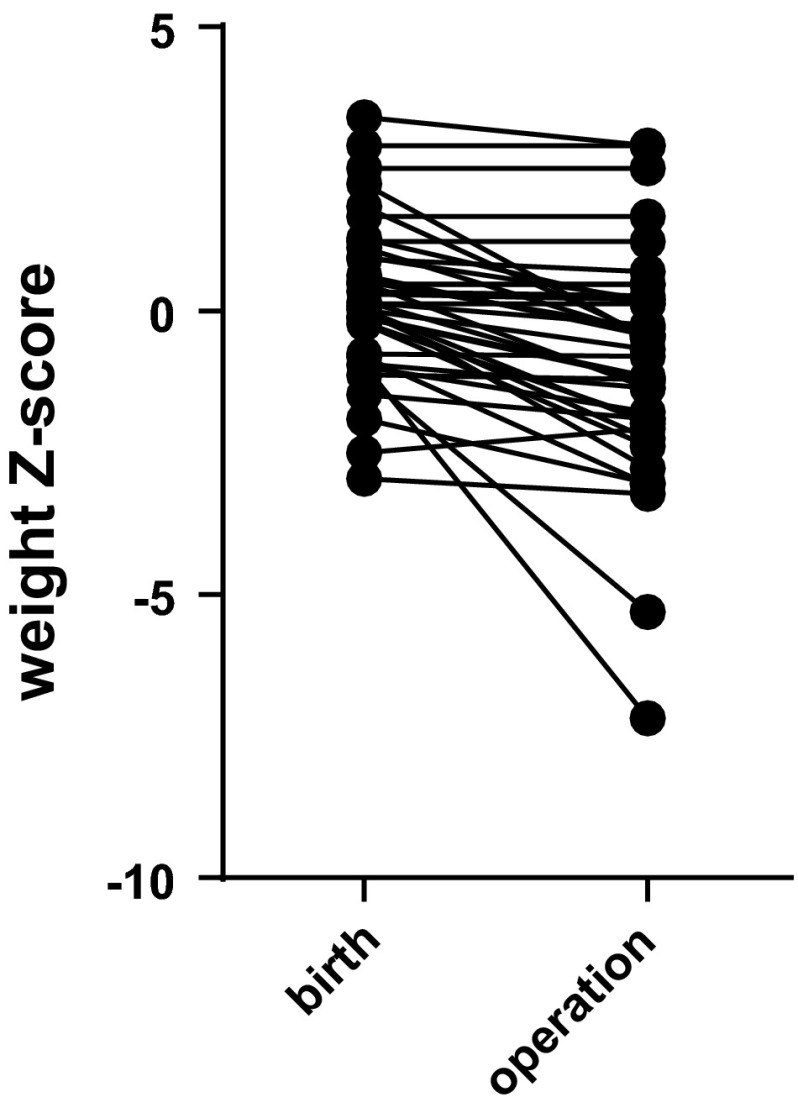
Weight *Z*-score at birth and at operation in infants undergoing ileostomy, colostomy, or cystostomy

Weight *Z*-score at birth was 0.18 ± 1.35, but infants lost weight so that by the time of operation, on day of life 8 (median, range 0–217), weight *Z*-score was −0.91 ± 2.00 (Fig. 1, *p* < 0.0001). Hence at operation, patients on average were slightly underweight but not malnourished; 31/40 (78 %) were normal according to WHO criteria, 4/40 (8 %) moderately malnourished and 5/40 (13 %) severely malnourished. Post-operatively, some patients subsequently became malnourished; 11 (28 %) were normal throughout follow-up, 15 (38 %) were moderately malnourished and 14 (35 %) severely malnourished according to WHO criteria. The demographics and clinical characteristics of these patients are shown in Tables 1, 2 respectively.

**Table 1:** Demographics of sample according to weight *Z*-score category

		All	Normal (*n* = 11)	Moderately malnourished (*n* = 15)	Severely malnourished (*n* = 14)	*p*
Gestation (weeks)		33 ± 4.86	36 ± 1.81	32 ± 6.06	31 ± 4.07	0.03
Gender	Male	22 (55 %)	3 (27 %)	9 (60 %)	8 (57 %)	0.20
	Female	18 (45 %)	8 (73 %)	6 (40 %)	6 (43 %)	
Age at surgery (days)		10 (0–220)	2 (0–220)	14 (1–87)	11 (1–212)	0.59
Diagnosis	Hirschsprung’s	4 (10 %)	0	0	4 (31 %)	
	Necrotizing enterocolitis	12 (30 %)	1 (8 %)	8 (53 %)	3 (23 %)	
	Imperforate anus	8 (20 %)	4 (33 %)	2 (13 %)	2 (15 %)	
	Malrotation	3 (8 %)	1 (8 %)	2 (13 %)	0	
	Meconium ileus	5 (13 %)	1 (8 %)	0	4 (31 %)	
	Neurogenic bowel disorders	2 (5 %)	2 (17 %)	0	0	
	Gastroschisis	1 (3 %)	1 (8 %)	0	0	
	Non-GI	5 (13 %)	2 (17 %)	3 (20 %)	0	
Surgery	Ileostomy	29 (73 %)	5 (45 %)	10 (67 %)	14 (100 %)	
	Colostomy	7 (18 %)	3 (27 %)	4 (27 %)	0	
	Cystostomy	4 (10 %)	3 (27 %)	1 (7 %)	0	

Normally distributed continuous data presented as mean ± (SD), non-normally distributed continuous data is presented as median (range). Categorical data is presented as *n* (%). Chi-square and ANOVA were used to produce *p*-values

*Non-GI* non-gastroenterological disease

**Table 2:** Clinical variables of sample according to weight *Z*-score category

	All	Normal (*n* = 11)	Moderately malnourished (*n* = 15)	Severely malnourished (*n* = 14)	*p*
Kidney disease	11 (28 %)	4 (36 %)	4 (27 %)	3 (21 %)	0.71
PO complications	14 (35 %)	4 (45 %)	4 (27 %)	6 (43 %)	0.65
PO infection	17 (43 %)	4 (36 %)	7 (47 %)	6 (43 %)	0.87
Diuretics	12 (30 %)	2 (18 %)	5 (33 %)	5 (36 %)	0.52
Length of admission (days)		36 (5–166)	32 (8–103)	44 (8–182)	0.51
NICU stay (days)		3 ± 4.69	4 ± 6.01	10 ± 10.37	0.05

Normally distributed continuous data presented as mean ± (SD), non-normally distributed continuous data presented as median (range). Categorical data presented as *n* (%). Chi square and ANOVA were used to produce *p*-values

*NICU* neonatal intensive care unit, *PO* post-operative

### Urinary sodium measurements

Of the 40 patients (all of whom had at least three urinary sodium measurements), only four patients (10 %) always had urinary sodium >30 mM, 6 (15 %) patients had their lowest recorded urinary sodium between 10 and 30 mM and 30 patients (75 %) had a recorded urinary sodium <10 mM at some time. The distribution of these urinary sodium measurements by WHO grade of malnutrition is shown in Table 3.

**Table 3:** Relationship between urinary sodium and degree of malnourishment

	Lowest urinary sodium		
	Normal (>30 mM)	Deficient (10–30 mM)	Very deficient (<10 mM)
Normal weight *Z* <2, *Z* >−2	2	3	6
Moderately malnourished *Z*<−2	2	2	11
Severely malnourished *Z* <−3	0	1	13
Total	4 (10 %)	6 (15 %)	30 (75 %)

Frequency of cases is presented as *n* (%)

### Relationship between electrolytes and growth

**Fig. 2:**
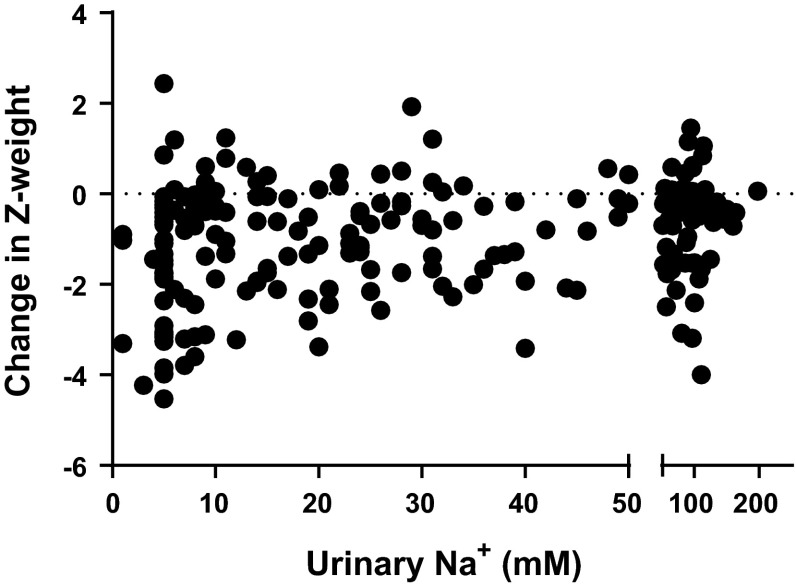
Effect of urinary [Na^+^] change in weight *Z*-score from operation to measurement. There was a significant relationship between urinary sodium and growth (Spearman Rank correlation coefficient *r* = 0.2432, *p* < 0.0003)

Electrolyte data were not normally distributed so that correlations between electrolytes and growth were tested using the non-parametric Spearman’s rank correlation coefficient. Urinary sodium levels positively correlated with change in weight *Z*-score (*r* = 0.2432, *p* < 0.0003, Fig. 2) so that lower urinary sodium values were associated with larger growth deficits. There was a weak correlation between urinary potassium and weight *Z*-score which failed to reach significance (*r* = 0.1165, *p* = 0.085), but there was no relationship between serum sodium or potassium and change in weight-*Z*-score.

**Fig. 3:**
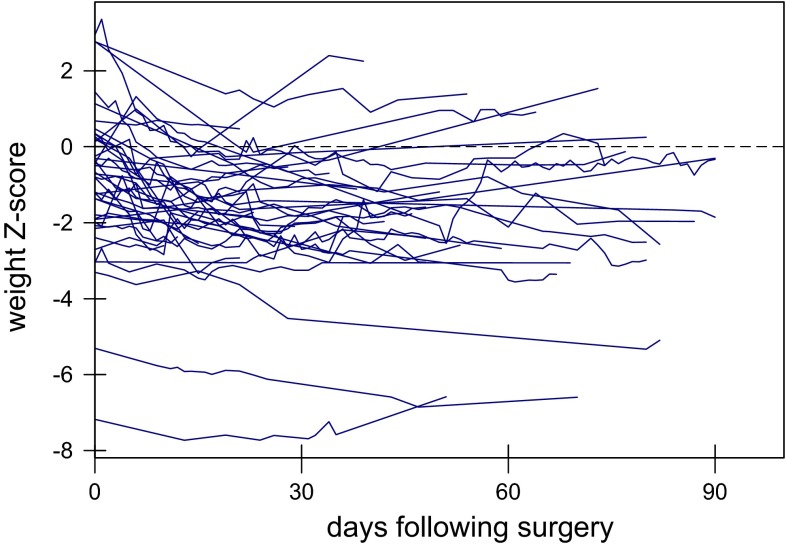
Longitudinal growth of studied patients

**Fig. 4:**
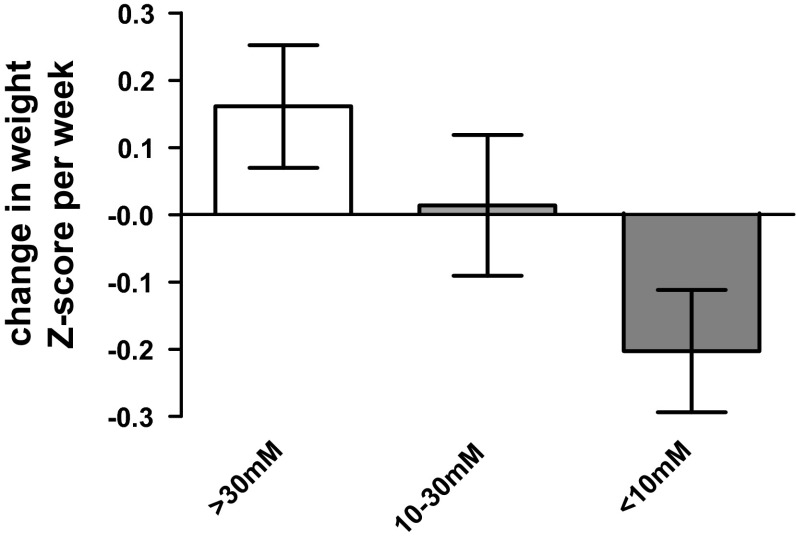
Estimates of growth from multilevel regression modelling. Patients were split into three groups based on lowest measured urinary sodium and change in weight *Z*-score per week postoperatively assessed by multilevel modelling; data are mean ± SEM

To determine the effects of low urinary sodium on longitudinal growth, we performed multilevel modelling. The growth of the patients over time is shown in Fig. 3. We first grouped patients into: (i) those who always had urinary sodium >30 mM and (ii) those whose urinary sodium measurements were between 10 and 30 mM and (iii) those that had urinary sodium <10 mM at some point. Multilevel modelling showed that on average, those with a urinary sodium always above 30 mM grew by +0.161 ± 0.091 *Z*-scores per week following surgery (*p* = 0.075), those with a urinary sodium between 10 and 30 mM grew by +0.014 ± 0.105 per week (*p* = 0.909) whereas those with a urinary sodium less than 10 mM at some time showed a growth failure by −0.203 ± 0.091 *Z*-scores per week (*p* = 0.027, Fig. 4). However, this analysis was limited by the small number of patients in the 10–30 and >30 mM urinary sodium groups. When these two groups were combined, patients >10 mM showed significant improvement in growth following surgery (+0.175 ± 0.056 *Z*-scores per week, *p* = 0.001) whereas those with a urinary sodium of less than 10 mM at some time had a significant growth failure, losing 0.21 ± 0.049 *Z*-scores per week (*p* < 0.0001).

## Discussion

The main finding of this study confirms the link between sodium and growth in surgical infants with a stoma. Of the patients studied, only ileostomy patients developed severe malnourishment, whereas patients with colostomy displayed both normal growth and moderate malnutrition; only one patient with cystostomy was moderately malnourished and the others were normal. Patients with NEC made up the majority of the diagnoses and were also more likely to develop moderate-severe malnourishment. These three procedures were chosen as patients all have repeated sodium measurements and were expected to display a range of both urinary sodium values and growth. The small bowel in the gastrointestinal tract is more likely to produce excretions that have not been sufficiently digested. This results in loss of dietary nutrients through the stoma bag. Excretions are usually liquid and contain high concentrations of electrolytes including sodium, chloride and bicarbonate ions. Surgical incision and handling of the gut can cause an immune response in the villous infrastructure that reduces absorption and potentiates loss of these electrolytes. Consequently, ileostomy patients are more likely to suffer from hyponatremia, metabolic acidosis and dehydration [2, 11]. This effect is seen less in colostomies because the large bowel absorbs most of the fluid in the gut including its electrolytes; excrement in colostomy stoma bags is usually more solid. The clinical characteristics of the group suggest that patients, who required more emergency support post-operatively, in the form of neonatal ICU stay, had a greater severity of malnutrition.

The main finding from this study is that urinary sodium levels appear to correlate with weight. From descriptive data alone, one can observe that post-operatively the majority of patients became both malnourished and severely deficient in urinary sodium levels. Although this is an association and not proof of causality, evidence from other studies suggests that there is indeed a causal link between sodium deficiency and poor weight gain in patients with a stoma.

Our findings broadly agree with those of Bower [2] who suggested that at least 10 mM urinary sodium is necessary for growth and more recently those of Butterworth et al. [7] who suggested optimum growth above 30 mM urinary sodium. Our study also suggests that optimum growth is observed above 30 mM urinary sodium, although patients with their lowest measured sodium level between 10 and 30 mM were exhibited better growth than those with their lowest measured sodium <10 mM. However, these studies, including our own, all suffer from the retrospective study design. Patients exhibiting poor growth are more likely to have urinary electrolytes measured, so these studies may over-report growth failure. In addition, a single time point urinary sodium measurement is not ideal for assessing whole body sodium adequacy. Thus, future studies should be performed to: (i) examine growth in a group of patients in whom urinary sodium is measured prospectively on a pre-determined schedule, and (ii) assess whether there is a better method to assess sodium adequacy, such as a urinary sodium/creatinine ratio, as used in cystic fibrosis patients [3]. This would hopefully allow evidence-based guidelines for sodium monitoring and supplementation to be established.

## References

[1] Haycock GB (1993). The influence of sodium on growth in infancy. Pediatr nephrol.

[2] Bower TR, Pringle KC, Soper RT (1988). Sodium deficit causing decreased weight-gain and metabolic-acidosis in infants with ileostomy. J Pediatr Surg.

[3] Coates AJ, Crofton PM, Marshall T (2009). Evaluation of salt supplementation in CF infants. J Cyst Fibros.

[4] Skillman J, Cole A, Slator R (2011). Sodium supplementation in neonates with pierre robin sequence significantly improves weight gain if urinary sodium is low. Cleft Palate-Craniofac J.

[5] Salmon AP, Finkel Y, Silove ED, Evans JA, De Giovanni JV, Wright JG, Booth IW (1989). Sodium balance in infants with severe congestive heart failure. Lancet.

[6] Nightingale J, Woodward JM (2006). Guidelines for management of patients with a short bowel. Gut.

[7] Butterworth SA, Lalari V, Dheensaw K (2014). Evaluation of sodium deficit in infants undergoing intestinal surgery. J Pediatr Surg.

[8] Pan H, Cole TJ (2012) LMSgrowth, a Microsoft Excel add-in to access growth references based on the LMS method, version 2.77. http://www.healthforallchildren.co.uk/

[9] Freeman JV, Cole TJ, Chinn S, Jones PR, White EM, Preece MA (1995). Cross sectional stature and weight reference curves for the UK, 1990. Arch Dis Child.

[10] de Onis M, Blössner M (1997) WHO global database on child growth and malnutrition. WHO, Geneva 10.1093/ije/dyg099 12913022

[11] Mews CF (1992). Topics in neonatal nutrition. Early ileostomy closure to prevent chronic salt and water losses in infants. J Perinatol.

